# Bayesian Network-Based Risk Analysis of Chemical Plant Explosion Accidents

**DOI:** 10.3390/ijerph17155364

**Published:** 2020-07-25

**Authors:** Yunmeng Lu, Tiantian Wang, Tiezhong Liu

**Affiliations:** Beijing Institute of Technology, School of Management and Economics, Beijing 100081, China; lu_yunmeng@163.com (Y.L.); 3120195835@bit.edu.cn (T.W.)

**Keywords:** chemical plant explosion accidents, Bayesian network, risk analysis, sensitivity analysis, emergency management

## Abstract

The chemical industry has made great contributions to the national economy, but frequent chemical plant explosion accidents (CPEAs) have also caused heavy property losses and casualties, as the CPEA is the result of interaction of many related risk factors, leading to uncertainty in the evolution of the accident. To systematically excavate and analyze the underlying causes of accidents, this paper first integrates emergency elements in the frame of orbit intersection theory and proposes 14 nodes to represent the evolution path of the accident. Then, combined with historical data and expert experience, a Bayesian network (BN) model of CPEAs was established. Through scenario analysis and sensitivity analysis, the interaction between factors and the impact of the factors on accident consequences was evaluated. It is found that the direct factors have the most obvious influence on the accident consequences, and the unsafe conditions contribute more than the unsafe behaviors. Furthermore, considering the factor chain, the management factors, especially safety education and training, are the key link of the accident that affects unsafe behaviors and unsafe conditions. Moreover, effective government emergency response has played a more prominent role in controlling environmental pollution. In addition, the complex network relationship between elements is presented in a sensitivity index matrix, and we extracted three important risk transmission paths from it. The research provides support for enterprises to formulate comprehensive safety production management strategies and control key factors in the risk transmission path to reduce CPEA risks.

## 1. Introduction

With the continuous development of the chemical industry, the production scale of chemical companies has gradually increased, resulting in frequent explosion accidents in chemical plants, causing heavy casualties and serious property losses, as well as great environmental pollution [1]. According to the accident data of the State Administration of Work Safety, from 2006 to 2015, there were 125 safety accidents in chemical companies in China, resulting in 542 deaths. Among them, 57 explosion accidents, accounting for 45.60% of the total number of accidents, caused a total of 301 deaths, accounting for 55.53% of the total number of deaths [2]. In recent years, there have also been many serious chemical plant explosion accidents (CPEAs). Two typical examples are listed as follows: On 21 March 2019, an explosion occurred at Tianjiayi Chemical Plant in Xiangshui County, Yancheng City, Jiangsu Province, resulting in 78 deaths and direct property losses of RMB 1.986 billion [3]. On 28 February 2012, Hebei Keeper Chemical Industries Co., Ltd., located in Zhao County, Shijiazhuang, Hebei, exploded. The accident caused a total of 25 deaths and 46 injuries, with a direct property loss of RMB 44.59 million [4]. Because chemical plants produce and store many kinds of flammable, explosive and toxic materials, once the explosion occurs, its destructive power is difficult to estimate, and can even lead to secondary derivative disasters [5]. Therefore, in order to prevent and effectively tap the risk of accidents and analyze the causes, the development and consequences of the accidents are very important.

The existing analysis methods of CPEAs are mainly conducted from two perspectives: statistical analysis and causation models. The statistical analysis summarizes the causes of accidents in terms of cause, type, time and region through accident case data. Li et al. conducted a statistical analysis of occurrence time, occurrence location, physical appearance, hazardous chemicals categories, occurrence stage and cause of accident, and found that the explosion accident in the production link accounted for the majority of cases [6]. Li et al. summarized and analyzed hazardous chemical accidents in 2018, and concluded that the production, storage, and transportation of hazardous chemicals are high-incidence links, and human factors dominate the accident factors [7]. Accident causation models can explain the mechanism of accidents and provide a theoretical framework for accident risk analysis and prevention [8]. Early accident causation models mainly analyze the occurrence of accidents from the perspective of human-based factors [9], such as accident prone tendency (APT), accident liability (AL), Surry’s model, Hale’s accident model, Lawrence’s accident model and so on. These models focus on the analysis of accident with single type of factors. In order to perform a comprehensive analysis of CPEAs and explore the deeper causes of the accidents, researchers introduced some more widely used models into the analysis of CPEAs, including the 24Model, the human factors analysis and classification system (HFACS), and fault tree analysis (FTA). Wang et al. used the 24 Model to research a typical hazardous chemical accident in China, and analyzed the causes of the accident from both individual and organizational levels [10]. Based on 102 accident cases, Wang et al. discussed the main manifestations of unsafe behavior, preconditions for unsafe behavior, unsafe supervision and organizational influence in chemical accidents, and improved the HFACS model to make it suitable for the chemical industry [11]. Wei et al. applied FTA and HFACS methods to explore the causal relationship of CPEAs. They found that FTA is helpful in analyzing the horizontal logical relationship between causes, and HFACS is more suitable for exploring the underlying causes of accidents [4]. To explore the logical relationship between factors and the development of accidents, Heinrich combined a series of accident factors to form an accident causal chain theory, namely Heinrich’s domino theory [12]. Based on this theory, some studies have proposed improved models. Bird’s accident causation model analyzes the underlying causes of unsafe behavior of humans and the unsafe state of things, and proposes that management defects are the root cause of accidents. Kitagawa’s accident causation model discovered that social factors played an important role in the accident. Orbit intersection theory, in describing the occurrence and development of accidents, emphasizes the development and connection of the human’s orbit and objects’ orbit, and reveals the development process of accidents. Orbit intersection theory can systematically excavate the underlying causes of accidents and is a powerful tool for investigating the causes of accidents. It is widely used in the analysis of oil explosion accidents, coal mine accidents and manufacturing safety production problems [13,14,15]. Because CPEAs are the result of interaction of many factors, the various stages of its occurrence and development involve many uncertainties, including internal and external factors. This paper uses the orbit intersection theory to systematically analyze the risk factors of CPEAs from the accident development causal chain.

In addition to the simple linear orbit cross-relationship between factors, the development of CPEAs also shows a complex network relationship that affects each other. In order to clarify the relationship between factors in depth, the establishment of a quantitative risk analysis model combined with risk factors and consequences is of great significance for preventing accidents. In the quantitative analysis of risk uncertainty, the probability-based model is an effective method, including the probability tree analysis method, Markov chain and Bayesian network [16]. However, the structure of the probabilistic tree analysis method is too simple, and it is difficult to model the complex network relationship among the factors. The Markov chain emphasizes the state transition between risks and lacks the analysis of scenarios under the combined action of multiple factors. The Bayesian network (BN) is a graphical network of probabilistic reasoning, and it can analyze the uncertain relationship between variables in a complex network [17]. This method is widely used in risk analysis [18,19,20,21], risk assessment [22,23,24,25] and decision-making [26,27]. Zhu et al. constructed a BN model of chemical terrorist attacks to conduct risk analysis, which provided theoretical support for the security prevention work of the risk management department [28]. Liu et al. applied BN to analyze the role of emergency organization elements in the evolution of flood Na-Tech events [29]. Zhang et al. applied BN to establish a single-phase grounding accident risk assessment model to explore the evolution of an accident from its cause to its potential consequences [30]. Scholars have also introduced BN to study CPEAs. Wei et al. analyzed the evolution process of secondary disasters caused by earthquake disasters in the chemical park, and transformed them into BN for scenario reasoning [31]. Zhu established a BN model of CPEAs, and found that equipment safety management and personnel safety training are the most important measures to prevent chemical plant explosion risks [32].

Based on the orbit intersection theory framework, this paper forms an evolution path from the cause, emergency response and consequences of the accident, and uses BN to model the complex relationship between the factors in the evolution path and the impact of the factors on the consequences. Firstly, based on the evolution path of the accident, the cases of CPEAs were studied, and 14 nodes were extracted. Then, considering that the CPEA data are limited and random, Huang’s [33] hybrid method is used to combine expert knowledge and historical cases to construct a BN to model CPEAs, namely BN-CPEAs, which quantitatively expressed the probability relationship between factors in CPEAs. Finally, scenario analysis and sensitive analysis are used to analyze the quantitative relationship between the factors and evaluate the contribution of these factors to the consequences of the accident. From this, we analyzed the key factors of accidents and extracted the main risk transmission path of the CPEAs. The research can provide practical support for the safety management of chemical plants.

## 2. Methods

**Figure 1:**
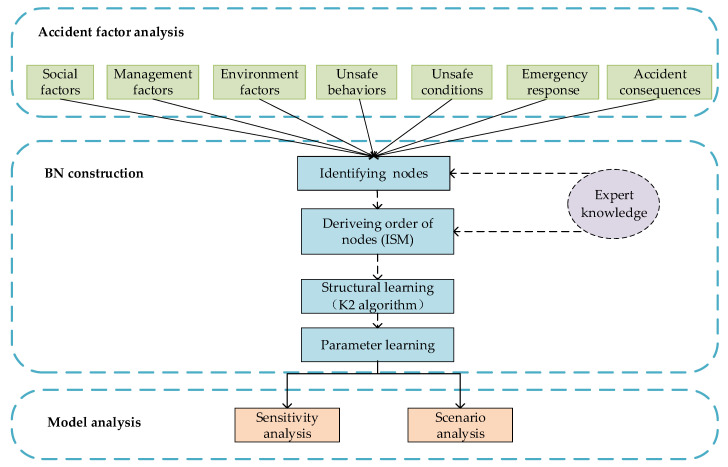
The overall structure of the method proposed in this paper.

For the analysis of the complex relationship between the various factors and the impact of these factors on the consequences of CPEAs, Figure 1 shows the overall structure of the method in this paper. The processing procedure mainly includes three steps from perspectives of qualitative and quantitative risk analysis: accident factor analysis, BN construction, and model analysis. The details are as follows:

(1) Considering the complexity of the cause of CPEAs, the method introduces orbit intersection theory to extract factors of CPEAs from unsafe behavior of humans, unsafe conditions of objects, management factors, emergency response and consequences.
(2) By constructing BN and using actual samples for structure and parameter learning, it provides model support for quantitative analysis of CPEAs. First, we encode the states of the extracted factors to quantify the data of actual CPEA cases. Then, a hybrid structure learning method “ISM-K2” is used to obtain the Bayesian network structure [33], thereby obtain the causal relationship between the variables. Finally, parameter learning is performed on the BN-CPEA model to determine the conditional probability distribution of the nodes.
(3) We use the learned BN model to quantitatively analyze the relationship between the factors of the CPEA and its impact on the accident, mainly including sensitivity analysis (SA) and scenario analysis.

### 2.1. Risks Analysis of CPEAs

The risk factors may exist in each stage of the occurrence and development of CPEAs, which makes the analysis of the factors of the accidents more complicated. This paper applies the orbit intersection theory and integrates the emergency elements of the accident to analyze the risk factors of the whole course of the accident to form an accident evolution path of accident causes, emergency response and consequences.

**Figure 2:**
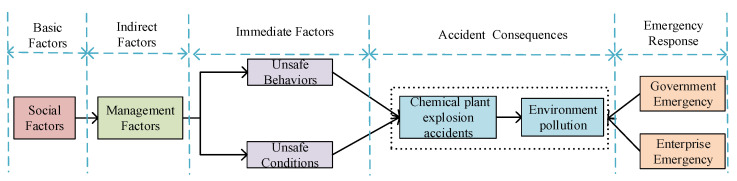
The occurrence and development process of chemical plant explosion accidents (CPEAs).

Orbit intersection theory is developed on Heinrich’s domino theory, which shows that accidents are the result of sequential development of many interrelated events [9]. The theory clarifies the various causes of accidents and the relationship between factors from five aspects: basic causes, indirect causes, direct causes, accident consequences and emergency response. Based on this theory, we analyze the occurrence and development of CPEAs, as shown in the Figure 2.

Social factors: the social factors are the basic causes of CPEAs. They are external factors of the enterprise. The external factor of CPEAs in this paper is mainly the safety supervision of relevant departments, such as safety supervision, environmental protection, quality supervision and other government agencies. Inadequate external supervision may lead to defects in enterprise safety management.

Indirect factors: management factors are indirect causes of CPEAs. They are internal factors of the enterprise, including enterprise safety management system, safety education and safety management organization [9]. When one or more of these factors are problematic, they often bring hidden dangers to production safety. Mismanagement can easily lead to unsafe behaviors and unsafe conditions.

Direct factors: the unsafe behavior of humans and the unsafe conditions of objects are the direct inducement of accidents, both of which will lead to accidents [7]. Due to the complexity of the production process, the requirements for equipment and personnel are extremely high. Equipment malfunction, lack of safety knowledge and skills of the operator, and operation errors may all lead to explosion accidents. Most accidents are due to the simultaneous occurrence of these two types of factors, and in many accidents, unsafe behaviors and unsafe conditions affect each other.

Emergency response: after an accident, emergency response, as a key link, usually requires the enterprise and the government to quickly organize rescue to reduce the losses caused by the accident [29].

Consequences: in terms of the consequences of accidents, the consequences are mainly quantified by property loss, casualties and environmental pollution [32]. CPEAs usually cause environmental pollution such as soil, water or atmosphere.

### 2.2. Bayesian Networks

In this paper, BN is used to quantitatively analyze the relationship between factors and the causal relationship between the event and factors in CPEAs. BN is a probabilistic network expressed as directed acyclic graph (DAG) and conditional probability table (CPT) [34]. In the network, the event and causing factors are random variables expressed as nodes, and their relationships are conditional probability expressed as directed edges. To construct BN, two conditions are needed. First, nodes and the directed edges should be determined to form a network structure. Second, the prior probability of each node of the known sample is completely counted [35].

In BN, DAG represents the structural relationship between variables through nodes and directed edges between the nodes. When a directed edge points from node *X*_1_ to node *X*_2_, then *X*_1_ is the parent node of *X*_2_, and *X*_2_ is the child node of *X*_1_. A node without a parent node is called a root node, and a node without child nodes is called a leaf node. The CPT in a DAG is a series of conditional probability distribution of nodes, and each of them can be expressed as the Bayesian formula:

(1) $$P\left(X_{2}|X_{1}\right)=\frac{P\left(X_{2}\right)P\left(X_{1}|X_{2}\right)}{P\left(X_{1}\right)}$$

where $P\left(X_{1}\right)$ and $P\left(X_{2}\right)$ are the prior probability of the parent node $X_{1}$ and child node $X_{2}$, and $P\left(X_{1}|X_{2}\right)$ and $P\left(X_{2}|X_{1}\right)$ are the prior and posterior conditional probability, respectively.

Thus, according to BN evidence theory, for each node $Y_{i}$, it is affected by all its parent nodes $Y_{1}$,$\;Y_{2},\ldots ,Y_{i-1}$, and its joint conditional probability distribution can be calculated according to the Bayesian formula, given as

(2) $$P\left(Y_{i}|Y_{i-1},\ldots ,Y_{1}\right)=P\left(Y_{i}|Paremts(Y_{i})\right)$$

where $Paremts(Y_{i})$ represents all the parent nodes of the node $Y_{i}$. Regarding the two nodes, they are independent of the joint conditional probability distribution of all their parent nodes [36]. As to a set of random variables represent as the nodes $\;\left\{X_{1},X_{2},\ldots ,X_{n}\right\}$, the joint probability distribution (JPD) is expressed as

(3) $$P\left(X\right)=\overset{n}{\underset{i=1}{\prod }}P\left(X_{i}|Paremts(X_{i})\right)$$

where *X* represents the set $\left\{X_{1},X_{2},\ldots ,X_{n}\right\}$.

Based on the necessary conditions of BN construction and the above analysis of the causes of CPEAs, the BN application in this paper contains three steps. First, the method summarizes the main factors of CPEAs from six aspects of unsafe behaviors, unsafe conditions, management factors, social factors, emergency response and consequences, and defines the nodes of the BN. Second, considering the large randomness of the CPEA data, the hybrid learning method called “ISM-K2” is introduced for BN structure learning. Specifically, we first combine expert experience to determine the pairwise influence relationship between BN variables, and then use the interpretive structure modeling method (ISM) model to obtain the node order; next, using the sorted nodes as input, we use the K2 algorithm to learn a complete BN structure. Finally, we learn the parameters using statistical samples to obtain the posterior probability of each node.

### 2.3. Sensitivity Analysis 

In the analysis of the causal factors of CPEAs, it is usually necessary to analyze the importance of each risk factor and put forward effective management suggestions and measures. In order to identify the key nodes in the network, this paper introduces a sensitivity index (SI) to perform SA [37,38,39]. SA is used to measure the sensitivity of a model’s output changes to changes of the model’s parameters. In BN, SA can quantify the degree of parameter changes of a node caused by changes in its lower-level nodes, which can determine which underlying factors contribute more to the upper-level factors in CPEAs.

The SI is expressed as the change rate of the probability of the resulting event caused by the change of the cause event, expressed as Formula (4).

(4) $$SI_{ij}=\frac{P\left(X_{j}=1/X_{i}=1\right)-P\left(X_{j}=1/X_{i}=0\right)}{P\left(X_{j}=1/X_{i}=0\right)}$$

where $X_{i}$ and $X_{j}$ are cause events and result events, respectively, $P\left(X_{j}=1/X_{i}=1\right)$ is the probability of occurrence of the consequence event when the cause event occurs, and $P\left(X_{j}=1/X_{i}=0\right)$ is the probability of occurrence of the consequence event when the cause event does not occur. The size of the sensitivity coefficient determines the impact on the output of the model. Therefore, by controlling the occurrence of the underlying event, the probability of the upper event is reduced.

## 3. Establishing a Bayesian Network

### 3.1. Identify BN Variables of CPEAs

In the analysis of CPEAs based on BN, the identification of the network’s nodes depends on the extraction of key factors of the accident. The investigation reports of CPEAs in China mainly summerize the accident based on the accountability system, which usually contains the direct and indirect factors of the accident, enabling us to trace the main influencing factors of CPEAs from these reports. Therefore, we use the risk analysis method mentioned in Section 2 to analyze the investigation reports and extract the main risk factors of CPEAs. We collected a total of 46 CPEA reports from the China Hazardous Chemicals Association and the Internet, and summarized the key elements in the development of the accidents. In addition, we also consulted the opinions of experts in the field of risk management, and finally identified 14 key nodes in terms of social factors, management factors, unsafe behaviors, unsafe conditions, emergency response, and accident consequences.

Subsequently, we encoded the state value of the node. Because many descriptions of the CPEA reports are not uniform, they usually have different degrees of information leakage in the description of various factors. We will look for the missing information of the case from the Internet and libraries. We first record the possible state of each node, and then discriminate the node state of each sample for a reasonable coding.

According to the above process, each node is defined as follows:

(1) Operation Error (A): Operation error refers to the unsafe behavior of workers in their actual operations, including operation violation and operation mistake. Operation violation means that the operator’s intentional failure to abide by the operation regulations results in wrong behavior. Operation mistake signifies unintentional wrong actions of operators, mainly including skill error and decision error. Skill error is an operation error caused by lack of job safety skills. Decision error is a mistake caused by emergency or negligence. Operation error is present in two states—if there is an operation error, it is defined as “present”, otherwise it is defined as “absent”.
(2) Attitude problem (B): Attitude problem mainly includes poor mental status and negative working status, such as employees leaving their posts without permission during working hours. If there is an attitude problem, it is defined as “present”, otherwise it is defined as “absent”.
(3) Design Defect (C): According to the Dangerous Chemicals Safety Management Regulations, design defect of chemical equipment endangers the safety of people’s lives and property. Design defects in the chemical plant equipment, such as rupture of the device, may cause hazardous materials to leak during the production process and may explode under certain conditions. Design defects are mainly manifested when the equipment quality does not meet the regulations or there is a safety risk. If there are design defects in the chemical plant equipment, it is defined as “present”, otherwise it is defined as “absent”.
(4) Improper use (D): In the process of using equipment in a chemical plant, configuration errors, expired service, overloaded equipment, or failure to carry out repairs and maintenance in time have major safety risks. In addition, the use of equipment must comply with the relevant regulations of the chemical plant industry. If the device is not used properly, the node is defined as “present”, otherwise it is defined as “absent”.
(5) Safety management system (E): Generally speaking, the chemical plant safety management system is the key guarantee for safe production. The safety management system problem mainly occurs when the production system is incomplete and not strict, and there may be loopholes in some positions or links, in addition to the system not being implemented according to regulations. When the above problem occurs, the node is defined as “unsound”, otherwise it is defined as “sound”.
(6) Safety management organization (F): The safety management organization of chemical enterprise refers to the administrative organization responsible for formulating safety production standards or supervising employees to perform safety responsibilities. If their work is not completed to the required standard, the node state is defined as “improper”. For example, lack of daily safety inspections, failure to supervise relevant personnel to correct hidden dangers in various links, and failure to perform safety production duties. Otherwise, it is defined as “proper”.
(7) Safety education and training (G): Safety education and training for chemical workers is the legal responsibility of the enterprise and the necessary production and operating conditions. Safety education and training can ensure that employees have the necessary safety production knowledge, are familiar with relevant safety production regulations and safety operation rules, and master the safety operation skills of this position. The state of this node is defined as “inadequate” or “adequate”.
(8) Illegal business (H): This refers to the violation of regulations in the production process of the enterprise, such as illegal production without obtaining production qualifications, or illegal storage of hazardous chemicals without approval. This node has two states—“present” and “absent”.
(9) Production environment (I): Generally speaking, the production environment problem refers to the factory construction or workshop design that does not comply with the established rules and regulations, such as the lack of protective design of the circuit and the unclear division of functional areas. The production environment has two states—“good” and “bad”.
(10) External supervision (J): External supervision mainly refers to the safety supervision of chemical enterprises by relevant departments. It is a social factor. The lack of external supervision may lead to problems in the implementation of internal safety management work in the enterprise. The most typical example is insufficient safety training for workers. The external supervision node is defined by two states—“inadequate” and “adequate”.
(11) Accident Severity (K): According to the Chinese classification standard Production Safety Accident Investigation and Handling Rules, the severity of accidents is determined according to the number of casualties and economic losses. An accident that causes more than 3 deaths, or more than 10 serious injuries, or direct economic losses of more than RMB 10 million is defined as a severe accident. Otherwise, it is a general accident. The states of the corresponding nodes are “severe” and “general”.
(12) Government emergency (L): According to practical experience, government emergency has two states: an efficient emergency and an inefficient emergency. An efficient government emergency response means that the official organization responds in a timely manner after the accident, and organizes rescue activities efficiently; an inefficient government emergency means that the official organization may not fully understand the situation on the spot, fail to organize a strong professional rescue team in time, or have poor command.
(13) Enterprise emergency (M): After the accident, chemical companies may miss the best rescue time, or lack of emergency plan drills, leading to chaos in the rescue organization. These are inefficient emergency responses. Conversely, an enterprise’s efficient emergency response means that the enterprise responds in a timely manner and the emergency work is professional and orderly.
(14) Environment pollution (N): CPEAs may lead to the leakage of harmful chemicals, resulting in environmental pollution of air, water and soil. This node has two states— “present” and “absent”.

Among these 14 nodes, “operational error” and “attitude problem” belong to the unsafe behavior of humans; “design defects” and “improper use” belong to unsafe conditions of things; “production environment” is an internal environment factor; “safety management system”, “safety management organization”, “safety education and training” and “illegal operation” are internal management factors of the enterprise; “external supervision” is an external social factor. The above factors are summarized as the causal factors of the accident. Emergency response after the accident includes “government emergency” and “enterprise emergency”, and the consequence of the accident is measured by “accident severity” and “environmental pollution”.

### 3.2. BN Structure Learning of CPEAs

Due to the large randomness of CPEA data, this paper adopts the ISM-K2 method, which first uses ISM to rank the nodes to obtain the overall order relationship among the nodes [40]. Then, the K2 algorithm is used to perform greedy hill climbing search and calculate the posterior probability of the subsequent nodes of a node, and to select the nodes with the largest posterior probability as its parent nodes according to the preset maximum number of parent nodes [41]. It can effectively integrate expert knowledge to improve the effect of network structure learning and obtain a more reasonable network model. In addition to better learn the BN structure, we use the bootstrap method to expand the sample data to 1000.

The implementation steps of BN structure learning of the CPEAs in this paper based on the ISM-K2 method are as follows:

(1) Conduct a questionnaire survey on three experts in the risk field to obtain the pairwise influence relationship between BN node variables.
(2) Based on the causal relationship between nodes, an adjacency matrix is constructed.
(3) Calculate the reachable matrix from the adjacency matrix.
(4) Divide the reachable matrix into different levels and obtain the order of the nodes.
(5) The ranking information obtained by the ISM method is used as the input of the K2 algorithm, and the maximum posterior probability is used as the evaluation index. Using the expanded samples, each node is searched for the variables with the highest scores to determine its parent variables.
(6) Based on the parent–child relationships between nodes obtained by the search method, construct the BN-CPEA structure.

**Figure 3:**
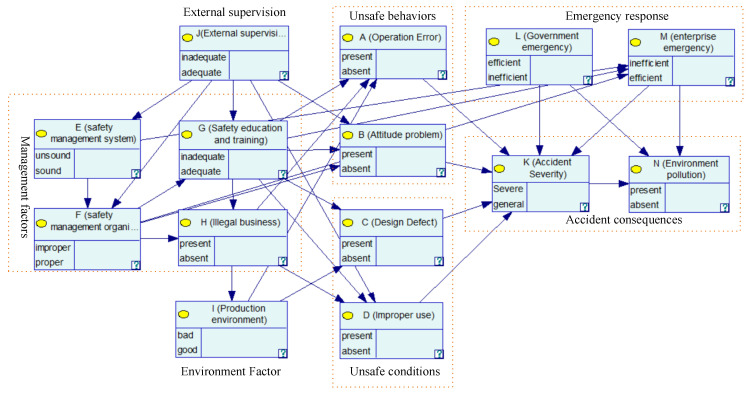
Initial Bayesian network (BN) structure for CPEAs.

Through ISM, the nodes are divided into nine levels: L9 = {J}, L8 = {E}, L7 = {F}, L6 = {G}, L5 = {H}, L4 = {I}, L3 = {A, B, C, D, L, M}, L2 = {K} and L1 = {N}, and the ISM-K2 algorithm is implemented using MATLAB. The structure of the obtained BN-CPEAs is shown in Figure 3.

In this structure, the inadequacy of the external supervision as a basic factor will lead to defects in enterprise safety management, and the defects in enterprise safety management, as indirect factors will cause unsafe behavior of humans and unsafe conditions of objects. Moreover, the unsafe behavior of humans and unsafe conditions of objects are the direct causes of accidents, which directly lead to accidents. The BN structure basically conforms to the orbit intersecting theory of accident.

### 3.3. BN Parameter Learning of CPEAs

Based on the BN-CPEA structure and training data, we use the EM (expectation maximization) algorithm that comes with the GeNIe 2.0 software to perform parameter learning to obtain a conditional probability table (CPT) for each node [42]. Specifically, when the BN structure is known, the real data of CPEAs are used to count the prior probability of each node, based on which the joint probability distribution of each node of BN is estimated and the posterior conditional probability of the node is calculated. Because the descriptions of CPEA reports are not uniform, they usually have different degrees of information omission in the description of various factors, resulting in incomplete data. The EM algorithm can convert incomplete data into complete data and use the maximum likelihood estimation (MLE) method for parameter learning, which is suitable for BN-CPEA parameter learning in this paper. In the MLE parameter learning stage, given the sample data $D=\left\{z_{1},z_{2},\ldots ,z_{n}\right\}$, we need to find a set of Bayesian network parameters with the highest probability of occurrence, expressed as $\arg\underset{\theta }{\max}p(\theta |D)$, where $\theta =\left\{\theta_{1},\theta_{2},\ldots \right\}$ is a set of parameters to be learned. According to Bayesian theorem, p(θ|D)=p(θ)p(D|θ)/p(D), where D is known data, then  p(D) is a definite value, and in MLE estimation, the probability p(θ) of the parameter is considered as a fixed value. Therefore, the parameter learning is converted to find a set of parameters so that the probability of including the sample D in the probability distribution is the largest, that is, $\arg\underset{\theta }{\max}p(D|\theta )$. Due to the independence of each sample, the optimization object is

(5) $$\arg\underset{\theta }{\max}\overset{n}{\underset{i=1}{\prod }}p(z_{i}|\theta )$$

**Figure 4:**
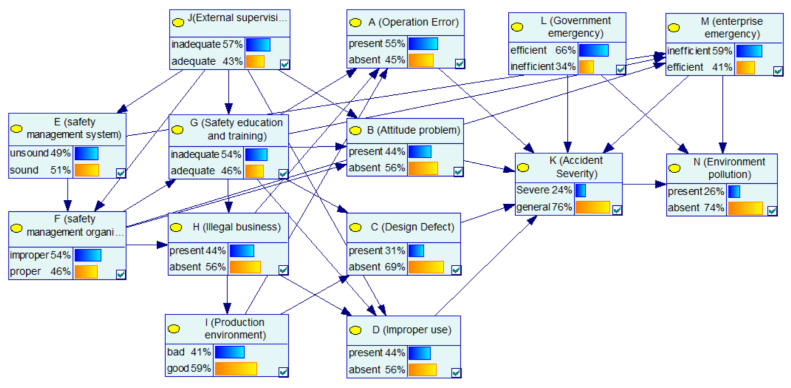
The learned BN-CPEA model.

After parameter learning, the BN-CPEA model is as shown in Figure 4. The conditional probability for each child node is also obtained. For simplicity, we only show the conditional probability tables (CPT) for the nodes in unsafe behaviors, as shown in Table 1 and Table 2. The learned BN model and conditional probabilities will be used to analysis the relationships and interactions between factors, and the effects of all factors on CPEA consequences.

## 4. Results and Discussions

### 4.1. Scenario Analysis

When a CPEA occurs, if the manager can obtain some actual information of the explosion accident in real time, the BN-CPEA model can be used to update the probability distribution of the corresponding node and analyze the posterior probability of other nodes based on the information. By analyzing the model, it can help enterprises improve the safety system and reduce the probability of accidents. We will analyze and discuss the model through two hypothetical scenarios.

#### 4.1.1. Case 1: The Basic Factors: Inadequate “External Supervision”

**Figure 5:**
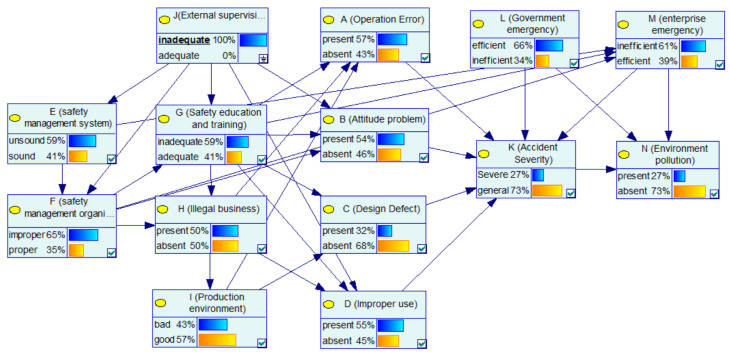
The posterior probability of other nodes when the state of the J node is inadequate.

**Figure 6:**
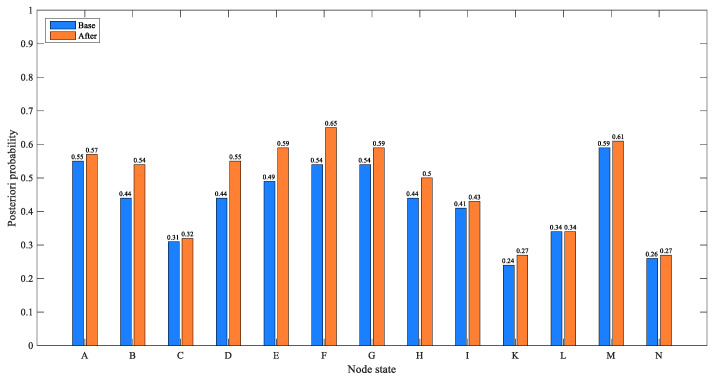
Comparison of the probability of other nodes before and after the state of “external supervision” changes to inadequate.

In the BN structure of this paper, “external supervision” as a root node has a certain impact on many nodes. Thus, this case focuses on analyzing the impact of “external supervision” on various factors and the severity of the accident. In Figure 4, after learning the network using sample data of the CPEAs, the priori probability of enterprise’s “safety management system” being unsound is 49%, and the priori probability of enterprise’s “safety management organization” being improper is 54%. The prior probability of “attitude problem” being present is 55%, and the prior probability of “improper use” of the equipment being present is 44%. Figure 5 shows the updated probability of each node when the “inadequate” state of the “external supervision” node is set to “100%”. Figure 6 shows the comparison of the probabilities of other nodes before and after this node state changes. In terms of safety management, the probability that the enterprise’s “safety management system” is unsound increases from the original 49% to 59%, with an increase of 10%. The probability that the enterprise’s “safety management organization” being improper is 65%, with an increase of 11%. The probability of the enterprise’s “illegal business” is 50%, with an increase of 6%. Additionally, in terms of unsafe behavior and unsafe conditions, the probability of the operator’s “attitude problem” is 54%, with an increase of 10%; the probability of the equipment “improper use” is 55%, with an increase of 11%. It can be seen that the probability of the aforementioned node state significantly increases, indicating that improper external supervision has a significant positive effect on factors such as unsound safety management system, improper safety management organization, illegal business, attitude problem, and improper use. The improper external supervision directly leads to an increased probability of problems with management factors, and indirectly leads to an increased probability of problems with factors related to unsafe behavior and unsafe conditions. This influencing relationship between factors is also in line with the view of orbit intersecting theory on factor transfer. In addition, for emergency response, the probability of an enterprise’s “emergency response” being “inefficient” increased by 2%. In addition, for the consequence event, the probability of “accident severity” being a “severe” state increased from 24% to 27%, indicating that external supervision has a certain impact on the severity of the accident. At the same time, the probability of environmental pollution increased by 1%. In general, as the basic factor of the chain reaction of CPEAs, external supervision will cause the occurrence of other factors and ultimately affect the occurrence and severity of the accident. Therefore, we should strengthen external supervision, improve the safety awareness of hazardous chemicals in the whole society, and avoid the harm caused by CPEAs.

#### 4.1.2. Case 2: Indirect Factors: Improper “Safety Management System” and Inadequate “Safety Education and Training”

**Figure 7:**
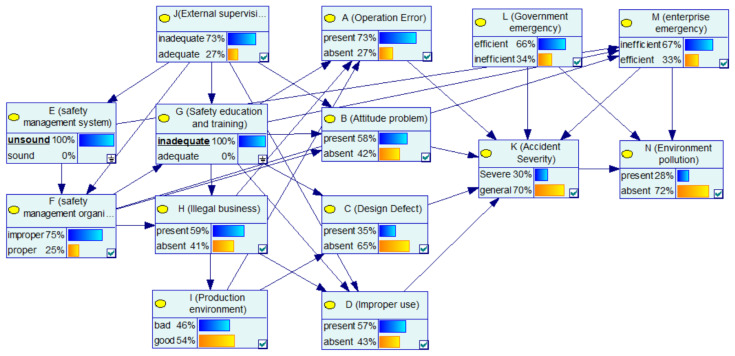
Case study for improper “safety management system” and inadequate “safety education and training“.

**Figure 8:**
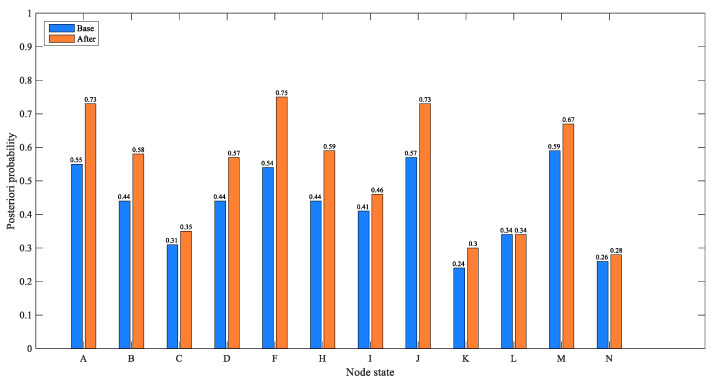
Comparison of the probability of other nodes before and after the states of “safety management system” and “safety education and training” change.

This case focuses on analyzing the role and impact of management factors in CPEAs. In Figure 4, the probability of “operation error”, “attitude problem”, “improper use” and improper “safety management organization” are 55%, 44%, 44% and 54%, respectively. When the enterprise has the both defects of “safety management system” and “safety education and training”, that is, the “unsound” and “inadequate” status of “safety management system” and “safety education and training” are set to 100%, respectively, the updated nodes‘ probabilities are as shown in Figure 7. Figure 8 shows the comparison of the probability of other nodes before and after the states of “safety management system” and “safety education and training” change to improper and inadequate. The probability of “ safety management organization“ being improper is 75%, with an increase of 21%; the probability of operating errors is 73%, with an increase of 18%; the probability of attitude problems is 58%, with an increase of 14%; the probability of improper use of equipment is 57%, with an increase of 13%; the probability of an enterprise’s poor emergency increased from 0.59% to 0.67%. In addition, the possibility of a severe CPEA increased by 6%, and the probability of environmental pollution increased from 0.26% to 0.28%. Combined with the analysis of the network structure, it is concluded that the safety management system affected the implementation of the safety management organization, and the enterprise’s safety management organization is responsible for safety education and training as well as inspection of equipment. Then, safety education and training directly affect the safety skills and safety awareness of the operators. This influential relationship makes the management factors an important part of the BN-CPEA model, playing a role of succession. It is worth mentioning that employees without safety education and training will greatly increase the improper use of equipments. Therefore, in the chemical plant production, it is necessary to further improve the enterprise safety management system, strengthen employee safety education and training, and improve the professional skills of employees, which will enable workers to operate tools safely and skillfully, so as to reduce operation errors, which can effectively reduce the probability of accidents.

### 4.2. Sensitivity Analysis of BN-CPEAs

CPEAs are the result of a chain reaction of many factors. Sensitivity analysis can perform uncertainty analysis on the changes of child nodes when the parent node (and the parent node of the parent node) changes in the BN-CPEA model to determine the relationship between the factors and which factors are the key factors of the accident. Thus, we can reduce the probability of upper-level events by controlling the occurrence of lower-level events, which helps to improve the corresponding corporate regulatory systems, prevention programs, and emergency plans to avoid accidents or reduce accident injuries. We will discuss it from the following three aspects.

#### 4.2.1. Sensitivity Index

**Figure 9:**
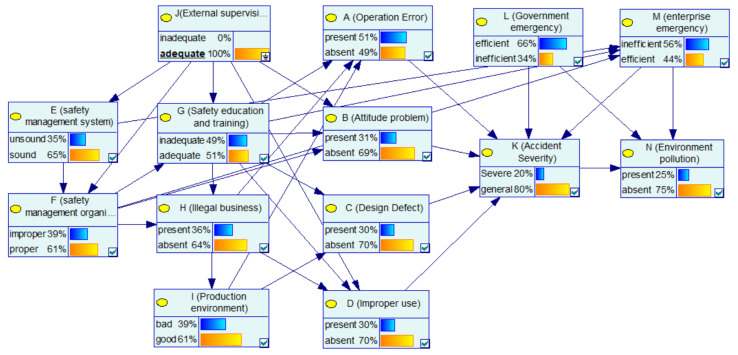
The posterior probability of other nodes when the state of the J node is adequate.

This section analyzes the SI between nodes in the BN model. First, through parameter learning, the posterior conditional probability of each node in the BN is obtained. Then, we change the probability of a node in BN-CPEAs, and calculate the SI of its upper node according to Formula (4). Taking the SI analysis of “attitude problem” caused by “external supervision” as an example, Figure 5 and Figure 9 show the changes in the probability of other nodes in the network before and after changes in state of the “external supervision” node. When the probability of inadequate external supervision is 100%, the probability of attitude problem is 0.54; when the “external supervision” is adequate, the probability of an “attitude problem” is 0.31. According to Formula (4), the SI of node B being present caused by node J can be calculated as 0.55 − 0.31/0.31 = 0.74.

According to the above operational approach, Table 3 shows the SI indicating the mutual influence between nodes. It can be seen from the table that the relationship between some factors of CPEAs is relatively close. For the unsafe behavior factors, the SI of node A to nodes G and H are 1.12 and 0.57, and the SI of node B to G and J are 0.81 and 0.74. For the unsafe condition factors, the SI of node C to node I is 1.05, and the SI of D to G, H, and J are 0.72, 0.57, and 0.83. In addition, the SI of node I to F and H are 0.53 and 1.22. For management factors, the SI of nodes E and F to node J are 0.69, 0.67, the SI of node F to node E is 0.79, and the SI of node H to nodes F and G are 2.94 and 0.56. In terms of emergency response, the sensitivity index of node M to nodes E and F are both 0.21. Taken together, the “attitude problem” of the operator and the “improper use” of equipment are more sensitive to “external supervision”, indicating that “external supervision” has an indirect impact on the attitude of the operator and the use of equipment. The “operation error”, “attitude problem” and “improper use” are more sensitive to “safety education and training” than other management factors, which reflects that “safety education and training” affects unsafe behavior and unsafe conditions. In addition, the company’s “illegal business” is very sensitive to the “safety management organization”, and the “production environment” is sensitive to whether the company is an “illegal business”. The results show that the company’s “safety management organization” and “illegal business” have an obvious impact on the “production environment”. In fact, the lack of safety management organization or the safety management organization work being inadequately implemented may lead to defects in production environment or safety management work, such as lack of improvements of production, or the production environment design not meeting the relevant standards, which may indirectly lead to accidents. This shows that the production environment is affected by management factors and is coupled with other risk factors, which indirectly leads to accidents. However, due to the high requirements on the production environment of chemical plants, an intelligent monitoring system can be used to analyze the actual environment in real time. Therefore, the results are consistent with reality.

In addition, we analyzed the impact of various factors on the accident consequences through SI. We found that the accident severity is more sensitive to unsafe conditions and unsafe behaviors. Specifically, the SI of the “accident severity” to “operation error”, “design defects”, and “improper use” are 1.07, 1.00, and 1.57, respectively. Compared with unsafe behavior, unsafe conditions have a greater impact on the accident. The sensitivity of the “accident severity” to the enterprise “safety management” is second. Among the management factors, the accident is the most sensitive to “safety education and training”, and its SI is 0.71. This shows that the direct factors have the most obvious influence on the accident consequences, and the unsafe conditions of objects contribute more than the unsafe behavior of humans. At the same time, “operation error”, “design defects “, and “improper use” are also greatly affected by the company’s “safety education and training”, which further illustrates that the enterprise’s safety education and training play an indispensable role in the accident. Therefore, strengthening enterprise safety education and training can indirectly reduce the losses caused by accidents, such as casualties and property losses. In addition, for “environmental pollution”, its SI to “government emergency” and chemicals plant “accident severity “are higher, respectively 1.16 and 1.58, which shows that the government’s timely and effective emergency can control environmental pollution to a certain extent.

In addition, based on the obtained sensitivity index matrix above and BN structure, the directional relationship between the factors with greater sensitivity is extracted, and finally points to the accident consequence to form risk transmission paths. It reveals the mutual influence of important management factors and the transmission relationship of their influence on direct factors and results. The main risk transmission paths extracted in this paper is as follows:

(1) Safety education and training→operation error→accident severity→environment pollution;
(2) External supervision→safety management system→safety management organization→illegal business→production environment→design defect→accident severity→environment pollution;
(3) Safety education and training→improper use→accident severity→environment pollution.

#### 4.2.2. Impact of the Emergency Response Measures

Fast and effective emergency response can effectively reduce the losses caused by CPEAs. Different organizations have their own roles in accident response. In response to CPEAs, the government and enterprises have played a very important role in the emergency response. In order to assess the impact of government and enterprise emergency response, we only changed the probability of these two nodes and designed four emergency response scenarios. The state settings of “government emergency” and “enterprise emergency” nodes are shown in Table 4.

According to the above four scenarios, by setting the state values of the two node variables of “government emergency” and “enterprise emergency” and updating the BN parameters, we obtained the posterior probability of the “accident severity” and “environmental pollution”. As shown in Table 4, comparing pattern 3 and 4, when both the government and the enterprise are in an efficient emergency, the probability of “accident severity” being a severe state is 20%, and the probability of environmental pollution is 15%. When both the government and the enterprise emergency display an inefficient emergency response, 12% of general accidents become severe accidents, and the probability of severe accidents becomes 32%, indicating that the inefficient emergency of the government and enterprise will cause some less serious accidents to become more serious. Because the severity levels of CPEAs are divided according to casualties and property losses, timely rescue operations can reduce casualties and property losses, which make it possible to quickly send casualties to receive medical treatment to avoid secondary injuries. In addition, the probability of environmental pollution caused by mode 3 is lower than mode 4 by 29%, and the decrease rate is nearly two-thirds. This shows that the effective response of both together plays an important role in controlling environmental pollution.

Comparing patterns 2 and 1, when switching from a government efficient emergency and an enterprise inefficient emergency to an enterprise efficient emergency and a government inefficient emergency, the probability of occurrence of severe CPEAs is reduced by 5%, and the probability of environmental pollution is reduced by 16%. This shows that the government’s efficient emergency plays a more important role than enterprises in controlling environmental pollutions, and the government’s performance is more prominent. The possible reason is that after the accident, the government can promptly mobilize relevant experts in the environmental field, promptly and accurately propose an emergency plan, and take effective control measures in a timely manner to prevent the leaked hazardous chemical materials from being exposed to the ecological environment such as soil and water, which finally reduce the probability of environment pollution.

#### 4.2.3. Evaluation of the Extreme Situations of CPEAS

As the production technology and process of hazardous chemicals become more complicated, the explosion of hazardous chemicals will have serious consequences. In order to simulate the extreme situation of such complex accidents, we only change the probability of the accident severity node to set the evidence of the most severe and ideal situations, and estimate the situation of other nodes. In the extreme cases, the initialization of the node states are as follows: (a) in the worst case, we set the probability of the “severe” state of the accident severity node to 100%; (b) in the most ideal case, we set the probability of the “general” state of the accident severity node to 100%.

**Figure 10:**
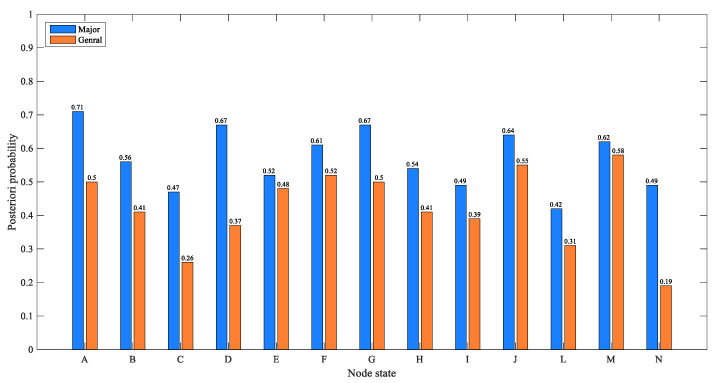
Estimated probabilities of the nodes under the most extreme situation of CPEAs.

Figure 10 shows the posterior probability distribution of nodes in two extreme cases. In general, when the two extreme cases are switched, the direction of the probability change of each factor is the same, reflecting that each factor has the same direction effect on the accident. In addition, in the most serious cases, the posterior probability of nodes A, D, G are relatively higher, and they are 0.71, 0.67, and 0.67 respectively, which indicates that the “operation error”, the “improper use” of equipment in unsafe conditions and the improper “safety education and training” in the management factors account for a relatively important proportion. When we changed the accident node to the most ideal situation, we found that some factors have more obvious probability changes than others, including “operational error”, “attitude problem”, equipment “design defect”, “improper use”, “safety education and training”, and “illegal business”, of which the first four factors are direct factors, and the latter two are management factors. Their posterior probability dropped by 21%, 15%, 21%, 30%, 17%, and 13%, respectively. This result shows that the probability of direct factors varies greatly in extreme situations, followed by management factors. In addition, the probability of an inefficient emergency for the government and enterprises fell by 11% and 4%, respectively, indicating that the more serious CPEAs are more likely to cause emergency failure and have higher requirements for emergency response. It is worth mentioning that the posterior probability of environmental pollution decreased by 30%, indicating that severe CPEAs are more likely to cause environmental pollution.

## 5. Conclusions

This paper integrates emergency elements under the framework of orbit intersection theory, and extracts 14 nodes from unsafe behavior, unsafe conditions, management factors, social factors, emergency responses and accident consequences. Combined with expert knowledge, the BN-CPEA model is obtained through structure learning and parameter learning. The main contributions and conclusions of this article are as follows:

(1) Through sensitivity analysis, the paper reveals the importance of the 12 factors to the accident severity and environmental pollution. Operation error, design defects, and improper use of equipment have the greatest impact on the accident severity among all factors. They are all direct factors and are largely affected by safety education and training. Furthermore, in management factors, the factor of safety education and training has the greatest effect on accident consequences. This shows that the direct factors have the most obvious influence on the accident consequences, and the unsafe conditions of objects contribute more than the unsafe behavior of humans. When considering the factor chain, it was also observed that strengthening the safety education and training of operators can effectively reduce operation errors and improper use of equipment, thereby significantly reducing the risk of accidents. In terms of environmental pollution, the government emergency response has played a prominent role in controlling it, and a timely and effective emergency response can reduce the risk of environmental pollution, property loss and casualties.
(2) The mutual influence between various factors is analyzed. We found that although safety management factors are indirect factors of CPEAs, these factors are important links in the accident occurrence and development chain. The management factors have an important impact on the unsafe behaviors, unsafe conditions, and production environment, and it also has a certain impact on the emergency response. Strengthening the management of humans and objects in chemical plants can effectively reduce the occurrence of direct factors of CPEAs, thereby avoiding accidents. Moreover, it helps to better organize emergency response after the accident. Furthermore, three important risk transfer paths have been extracted, which are of great significance for proposing mutually coordinated management strategies, such as making up for the defects of a certain factor by strengthening the role of other factors in the important transfer path.
(3) Through scenario analysis, the mutual relationship between factors and the consequences are further discussed. The social factors, as the basic factors, directly affect the internal safety management factors of the enterprise. From a long-term perspective, the weak external supervision causes the increase in safety management risks, which lie as hidden dangers for the safety production of the enterprise. In the indirect factors, management factors play a vital role in the whole network, and improper management eventually leads to the occurrence of unsafe behaviors and unsafe conditions. Unsafe behaviors and unsafe conditions will lead to accidents, and eventually affect property loss, casualties and environmental pollution.

The above conclusions can provide a reference for the actual production safety management of chemical plants. From the point of view of the importance of risk factors, strengthening the safety skills training of operators, regularly carrying out inspections of equipment safety and the hidden dangers in the production environment to reduce operational errors, improper use of equipment and design defects are important measures to prevent CPEAs. Enterprises should improve their safety management system, operate production legally, and ensure the implementation of safety management organizations. In addition, fast and effective emergency response can reduce the losses caused by accidents. Enterprises should formulate detailed emergency plans and regularly organize emergency drills. Relevant departments can establish an integrated emergency information management platform to provide assistance to the emergency rescue process according to the actual situation. Finally, external supervision is the external driving force for advancing the safety management of enterprises. Government agencies should strengthen safety supervision and inspection and formulate corresponding measures to ensure public supervision power. From the perspective of CPEAs’ important risk transmission path, by controlling the risk factors in the path, it can provide a reference for proposing coordinated management strategies. Moreover, although this paper mainly analyzes CPEAs in China, the cause framework of the proposed approach can flexibly integrate various elements according to the actual situation of governments and enterprises of various countries, and be applied to accident analysis in different countries and regions. It helps to put forward differentiated rectification plans and management suggestions.

Although the research results of this paper have certain reference significance for avoiding CPEAs, the research of this paper still has some limitations. First of all, the accident data in this paper mainly come from the accident investigation report, which makes it difficult for us to obtain more delicate factors such as technology, employment, age and quality of assets when extracting accident nodes. Secondly, richer data will help us analyze the importance and mutual relationship of accident causes more accurately. Finally, although the proposed method in this paper effectively combines expert experience and historical data, the evolution of accidents changes dynamically over time, but this paper does not cover more complex dynamic models. In future research, we will conduct interviews and investigations on the cases to obtain more abundant factors, and consider combining dynamic models to strengthen the risk analysis of CPEAs.

## Figures and Tables

**Table 1:** Conditional probability tables for the “operation error” node.

Nodes Status	G-Inadequate				G-Adequate			
	H-Present		H-Absent		H-Present		H-Absent	
	I-Bad	I-Good	I-Bad	I-Good	I-Bad	I-Good	I-Bad	I-Good
B-F	0.82	0.72	0.73	0.63	0.56	0.46	0.32	0.22
B-S	0.18	0.28	0.27	0.37	0.44	0.54	0.68	0.79

**Table 2:** Conditional probability tables for the “attitude problem” node.

Nodes Status	J-Inadequate				J-Adequate			
	G-Inadequate		G-Adequate		G-Inadequate		G-Adequate	
	F-Improper	F-Proper	F-Improper	F-Proper	F-Improper	F-Proper	F-Improper	F-Proper
A-F	0.63	0.52	0.51	0.41	0.52	0.47	0.23	0.12
A-S	0.37	0.48	0.49	0.59	0.48	0.53	0.77	0.88

**Table 3:** Sensitivity index matrix of nodes in BN-CPEAs.

	A	B	C	D	E	F	G	H	I	K	M	N
J	0.12	0.74	0.07	0.83	0.69	0.67	0.20	0.39	0.10	0.35	0.09	0.08
E	0.10	0.23	0.07	0.20	—	0.79	0.10	0.41	0.13	0.18	0.21	0.04
F	0.30	0.49	0.14	0.42	—	—	0.28	2.94	0.53	0.35	0.21	0.08
G	1.12	0.81	0.26	0.72	—	—	—	0.56	0.16	0.61	0.11	0.17
H	0.57	—	0.33	0.57	—	—	—	—	1.22	0.45	—	0.12
I	0.38	—	1.05	—	—	—	—	—	—	0.33	—	0.08
A	—	—	—	—	—	—	—	—	—	1.07	—	0.17
B	—	—	—	—	—	—	—	—	—	0.58	—	0.12
C	—	—	—	—	—	—	—	—	—	1.00	—	0.20
D	—	—	—	—	—	—	—	—	—	1.57	—	0.25
L	—	—	—	—	—	—	—	—	—	0.43	—	1.16
M	—	—	—	—	—	—	—	—	—	0.14	—	0.26
K	—	—	—	—	—	—	—	—	—	—		1.58

Note: rows indicate cause factors; columns indicate result events.

**Table 4:** Estimated probability of accident consequences under different emergency response modes.

Posteriori Probability	Government Emergency	Enterprise Emergency	K-Severe	N-Present
Pattern 1	Efficient	Inefficient	0.22	0.21
Pattern 2	Inefficient	Efficient	0.27	0.37
Pattern 3	Efficient	Efficient	0.20	0.15
Pattern 4	Inefficient	Inefficient	0.32	0.44
